# *Haemophilus parasuis* VtaA2 is involved in adhesion to extracellular proteins

**DOI:** 10.1186/s13567-019-0687-2

**Published:** 2019-09-23

**Authors:** Mar Costa-Hurtado, Laura Garcia-Rodriguez, Sergi Lopez-Serrano, Virginia Aragon

**Affiliations:** IRTA, Centre de Recerca en Sanitat Animal (CReSA, IRTA-UAB), Campus de la Universitat Autònoma de Barcelona, 08193 Bellaterra, Spain

## Abstract

*Haemophilus parasuis* is part of the microbiota of the upper respiratory tract in swine. However, virulent strains can cause a systemic disease known as Glässer’s disease. Several virulence factors have been described in *H. parasuis* including the virulence-associated trimeric autotransporters (VtaAs). VtaA2 is up-regulated during infection and is only found in virulent strains. In order to determine its biological function, the *vtaA2* gene was cloned with its native promotor region in pACYC184, and the transformed *Escherichia coli* was used to perform functional in vitro assays. VtaA2 was found to have a role in attachment to plastic, mucin, BSA, fibronectin and collagen. As other VtaAs from *H. parasuis*, the passenger domain of VtaA2 contains collagen domains. In order to examine the contribution of the collagen repeats to VtaA2 function, a recombinant *vtaA2* without the central collagen domains was obtained and named *vtaA2OL*. VtaA2OL showed similar capacity than VtaA2 to adhere to plastic, mucin, BSA, fibronectin and plasma but a reduced capacity to adhere to collagen, suggesting that the collagen domains of VtaA2 are involved in collagen attachment. No function in cell adhesion and invasion to epithelial alveolar cell line A549 or unspecific binding to primary alveolar macrophages was found. Likewise VtaA2 had no role in serum or phagocytosis resistance. We propose that VtaA2 mediates adherence to the host by binding to the mucin, found in the upper respiratory tract mucus, and to the extracellular matrix proteins, present in the connective tissue of systemic sites, such as the serosa.

## Introduction

*Haemophilus parasuis* is a Gram-negative bacterium of the *Pasteurellaceae* family. It is an early colonizer of the upper respiratory tract and part of the normal microbiota of healthy pigs [1]. Under certain circumstances, virulent strains of *H. parasuis* can produce Glässer’s disease, which is characterized by fibrinous polyserositis, including arthritis, and meningitis [2]. Glässer’s disease represents a big economic burden for the pig industry [3] and prevention and control continue to be challenging since the pathogenicity of *H. parasuis* is not fully characterized.

Comparison between virulent and non-virulent strains allowed the description of some mechanisms of the pathogenesis of *H. parasuis*. Strains of *H. parasuis* display mechanisms for mucosal colonization by adhering and invading epithelial and endothelial cells [4–6]. *H. parasuis* virulence is associated to evasion of the innate immune system by degradation of IgA [7], resistance to phagocytosis by alveolar macrophages [8] and resistance to serum complement killing [9]. Thus, *H. parasuis* is able to reach internal organs, where it replicates in serosal surfaces. Presence of systemic bacteria produces a strong inflammation in the host [10]. Massive fibrin exudates, increased permeability by disruption of adherens junctions, invasion of endothelial cells, and unbalanced inflammatory response are probably key steps in the development of systemic disease, which causes the characteristic lesions of Glässer’s disease, including meningitis [10–13].

Antimicrobial treatment remains useful to control Glässer’s disease at the farm level. However, the rise of antimicrobial resistances is a serious threat to animal and public health. Global efforts have been adopted to reduce the use of antibiotics in veterinary medicine. Besides, the use of antimicrobials may affect the colonization by *H. parasuis* and modify disease dynamics and the development of immune responses [14–16], which may finally affect the immunocompetence of the host.

Virulence-associated trimeric autotransporters (VtaA) are surface exposed proteins of the Type V secretion system involved in host–pathogen interaction [17, 18] and promising vaccine candidates [19]. Trimeric autotransporters are composed of an N‐terminal signal peptide for transport across the inner membrane; a C‐terminal translocator domain that creates a pore with a β‐barrel structure in the outer membrane; and a passenger domain that is secreted to the bacterial surface [20]. The passenger domains of all the VtaAs possess motifs and repeats characteristic of adhesins, hemagglutinins and invasins, with various centrally located copies of triple helix collagen-like repeats [17]. Virulent *H. parasuis* strains have several copies of *vtaA* in their genome and some of them have been found to be immunogens [17, 21], demonstrating that these proteins are expressed during infection, and supporting their role in infection. Some *vtaA*s have been associated specifically to virulent strains [17, 22, 23], but so far a function has been assigned only to VtaA8 and VtaA9, with a role in phagocytosis resistance [18]. Through transcriptomic studies, *vtaA8* and *vtaA9* (together with other *vtaA*) were found to be up-regulated during lung infection [24]. Likewise, up-regulation of *vtaA*2 transcription in lung was detected after 2 h and after 1, 2 and 3 days post-infection [24], indicating that VtaA2 is involved in for lung infection.

VtaA2 comprises 1225 amino acids and presents a slightly different structure compared to VtaA8 and VtaA9. Although they display the same basic structure, the number of LbR-YadA-like repeats and specially the number of collagen repeats differ between VtaA2 and those found in VtaA8 or 9 [17, 25], suggesting a different function.

In the present study, we evaluated the function of VtaA2 by cloning *vtaA2* from the virulent strain Nagasaki of *H. parasuis* with its native promotor in an *E. coli* background. In vitro assays showed that VtaA2 has adhesion features that could be important for host infection.

## Materials and methods

### Bacterial strains and plasmids

Plasmids used in this work are indicated in Table 1. Plasmid pACYC184 is a low copy number vector, at about 15 copies per cell, and contains the p15A origin of replication. The unique BamHI site in the plasmid was used as cloning site.

**Table 1:** **Plasmids used in this work and their main characteristics**

Plasmids	Description	Reference
pACYC184	low copy number, CmR, TetR, 4245 bp	ATCC number 37033
pLGR-vtaA2	pACYC184 with a 4016 bp insert, including *vtaA2* and its promotor region	This work
pLGR-vtaA2OL	pACYC184 with a 2514 bp insert, including *vtaA2OL* and the promotor region of *vtaA2*	This work
pEGFP	*gfp*, AmR	Clontech

Cm: chloramphenicol, Tet: tetracycline, Am: ampicillin, R: resistant.

*Escherichia coli* BL21 (DE3) was used as the host for recombinant plasmids and was grown in Luria–Bertani (LB) medium, supplemented with 30 µg/mL chloramphenicol (Cm30).

### Cloning of *vtaA2* and protein expression

A fosmid genomic library of *H. parasuis* virulent strain Nagasaki previously produced [18] was used for *vtaA2* amplification. In order to detect clones containing the individual *vtaA* genes, a specific PCR previously described was used [23]. To identify fosmids carrying *vtaA2*, a PCR to detect a *vtaA2* fragment was performed with primers HPNK_01698_F and HPNK_01698_R (Nagasaki genome number ANKT01000000; Table 2) [24]. Gene *vtaA2* was detected in 2 fosmids and one of them was used as template to PCR-amplify *vtaA2* with primers BamHI-vtaAF and BamHI-vtaAR [18] and Platinum™SuperFI™ DNA polymerase (Invitrogen, Barcelona, Spain) (Table 2). Amplicon size was confirmed and the product cloned into pACYC184 to produce pLGR-vtaA2. Plasmid pLGR-vtaA2 was introduced in electrocompetent *E. coli* BL21 (DE3).

**Table 2:** **Primers used in PCR in this work**

Primer	Primer sequence (5′–3′)
HPNK_01698_F	GGAGTCATGCTCAACAGGCT
HPNK_01698_R	CAGCCTCTTCCTTCCTTCCG
BamHI-vtaAF^a^	GCGCGGATCCTCTTAGTTTTGTGTAACTCTTG
BamHI-vtaAR^a^	GCGCGGATCCTTCTAATTTATAGGTGCTAGATTAC
OL-vtaA2F	TGGCGATCTAGGACCTACGGGTCCAACGGG AC
OL-vtaA2R	CCGTAGGTCCTAGATCGCCATTTAGTTCATT
vtaA2-865F	AGCAGTGGCAAGGTAGGC
vtaA2-2655R	CTCCATCGGTAAACCGCTG

^a^BamHI site in primers is underlined.

In order to determine the role of the collagen-like repeats present in the passenger domain of VtaA2, a recombinant VtaA2 without the central protein fragment containing the collagen domains was produced. The 5′ terminus and 3′ terminus of the gene were amplified from the fosmid containing the *vtaA2* with overlapping primers (Table 2). The PCR fragment containing the promoter region and nucleotides 1 to 1050 of *vtaA2* (amplified with BamHI-vtaAF and OL-vtaA2R) and the second fragment containing nucleotides 2551 to 3678 of the *vtaA2* gene (amplified with OL-vtaA2F and BamHI-vtaAR) were purified and ligated, yielding pLGR-vtaA2OL. To confirm that the two gene fragments were in-frame, primers vtaA2-865F and vtaA2-2655R (downstream and upstream of the deleted gene section, Table 2) were used for sequencing.

Expression of the corresponding protein was assessed in the clones. *E. coli* BL21 containing pLGR-vtaA2 or pLGR-vtaA2OL were grown in LB-Cm30 up to late-logarithmic phase. Bacteria were pelleted and protein extraction was performed with TRIzol™ Reagent (Invitrogen) according to the manufacturer’s instructions. Protein extracts were quantified by the BCA protein assay kit (Pierce-Thermo Fisher Scientific, Sant Cugat del Vallès, Spain), loaded in a NuPAGE^®^ Novex 4–12% Bis–Tris Gel, followed by a transfer to a Hybond™ ECL™ nitrocellulose membrane (GE Healthcare, Barcelona, Spain). After blocking with 1% casein 0.05% Tween20 in PBS, membranes were incubated with the monoclonal antibody 69C6 [18] at 2 ng/µL in blocking solution. Proteins VtaA2 and its truncated version VtaA2OL were detected by chemiluminescence after incubation with peroxidase-conjugated goat anti-mouse immunoglobulin (Sigma-Aldrich, Madrid, Spain) diluted 1:10 000.

### Attachment assays

The role of VtaA2 in attachment was evaluated on plastic and on different protein substrates. Various time points (6 h, 12 h, 24 h, 48 h) were tested under static or shacking conditions. Attachment assays were performed in 96-well plates and attachment was measured by crystal violet staining as previously described with some modifications [26]. First, flat-bottom 96-well polystyrene plates (SPL, Life Sciences, Naechon- Myeon, Korea) were coated with 200 µL of 25 µg/mL mucin (Sigma Aldrich), fibronectin (Invitrogen) or collagen (type I, Sigma Aldrich) in carbonate-bicarbonate buffer (pH 9.6) in triplicate. Porcine plasma was also used. Plasma was obtained by centrifugation of heparinized blood from a healthy animal and used for coating at 1:2 or 1:10 dilution. Sampling was performed under institutional authorization and followed good veterinary practices. According to European (Directive 2010/63/EU) and Spanish (*Real Decreto* 53/2013) normative, this procedure did not require specific approval by an Ethical Committee. Bovine serum albumin (BSA; Sigma Aldrich) was used as non-specific protein control and some wells remained uncoated. After overnight incubation at 4 °C, wells were washed once with sterile PBS. Bacterial suspensions were prepared in PBS (OD_600_ of 0.3) using overnight agar cultures on LB-Cm30 of *E. coli* BL21 pACYC184 (negative control), pLGR-vtaA2 and pLGR-vtaA2OL. Bacterial suspensions were diluted down 1:100 in fresh LB broth with Cm30 and were used to inoculate wells in triplicate (100 µL/well).

Plates were incubated in static conditions or under shaking (100 rpm) for different time-points (3 h, 6 h, 12 h and 24 h). After incubation, plates were washed once by water immersion and attached bacteria were stained with 0.1% (w/v) crystal violet for 2 min. After three washes in water by immersion, plates were allowed to dry at 42 °C. Stain in the wells was solubilized with 70% ethanol and the released dye was quantified by measuring the absorbance at 590 nm with a microplate reader (Power Wave HT Microplate Spectrophotometer, BioTek, Winooski, USA).

### Cellular adhesion and invasion assays

Epithelial cell line A549 from human lung carcinoma (ATCC^®^ CCL-185™) was used for cell adhesion and invasion experiments. Cells were seeded in 96-well plates (SPL, Life Sciences) at a concentration of 6 × 10^4^ cells/well in Dulbecco’s modified Eagle’s medium (DMEM; Hyclone GE Healthcare) supplemented with 10% fetal bovine serum (FBS) and 1% l-glutamine; complete DMEM (CDMEM). Plates were incubated overnight at 37 °C with 5% CO_2_.

Bacterial suspensions of *E. coli* BL21 pACYC184 and *E. coli* BL21 pLGR-vtaA2 were prepared to reach an OD_600_ of 0.3 (Spectrophotometer VIS7200), which corresponded approximately to 10^8^ CFU/mL. Protein expression under conditions was confirmed by immunoblot as described above. Bacterial concentration was confirmed by serial dilution and plating. Duplicate wells with epithelial cells were inoculated with 100 µL of each bacterial suspension and plates were incubated at 37 °C with 5% CO_2_ for 1 h. After incubation, wells were washed three times with PBS to eliminate unbound bacteria. To release bacteria, epithelial cells in each well were lysed with 100 µL of sterile Milli-Q water and scrapped. Bacteria were counted by plating serial dilution of the lysates. This assay was performed twice.

To test cell invasion, the adhesion assay was performed as above for adhesion but with an additional step after the 1 h incubation to allow bacteria to invade the cells. The additional step consisted of 2 h incubation with 5 µg/mL penicillin G and 100 µg/mL gentamicin to kill extracellular bacteria. After washes, cells were lysed and bacteria were counted as in the standard adhesion procedure.

### Interaction with porcine alveolar macrophages (PAMs)

The ability of the *E. coli* clone carrying VtaA2 to attach to PAMs was also tested. All procedures involving animals followed EU and Spanish normative (Directive 2010/63/EU and *Real Decreto* 53/2013). PAMs were collected from bronchoalveolar fluid lavages from healthy pigs that were euthanized for this purpose following good veterinary practices. According to European (Directive 2010/63/) and Spanish (*Real Decreto* 53/2013) normative, this procedure did not require specific approval by an ethical committee. PAMs were isolated and stored at −150 °C following standard procedure [8].

Cell adhesion assays to PAMS were performed on ice to avoid active phagocytosis. Cells were thawed, gently washed of cryopreservant and seeded in 96-well plates at a concentration of 5 × 10^5^ cells per well. Plates were incubated at 37 °C with 5% CO_2_ overnight to allow the attachment of the cells to the wells. Bacterial suspensions were made in CDMEM (OD_600_ of 0.3) and 100 µL of these suspensions were used to inoculate triplicate wells. After a centrifugation during 10 min at 100 × *g* plates were incubated on ice for 1 h. Bacterial concentration in the initial inoculum was confirmed by dilution and plating. After incubation, PAMs were washed three times with PBS to eliminate unbound bacteria, and 100 µL of 0.1% saponin (Sigma Aldrich) in PBS was added to the wells to lyse the PAMs. Serial dilutions were plated in LB agar-Cm30. The assay was repeated three independent times.

Phagocytosis assays were performed as described before [8]. PAMs were seeded in 6-well plates at a concentration of 5 × 10^5^ PAMs/well in CDMEM. After incubation to allow attachment of the PAMs, (overnight incubation) wells were inoculated with bacteria at a multiplicity of infection (MOI) of 200. *E. coli* clones were labelled using different strategies to ensure consistent fluorescence of all the bacterial population, using fluorescein isothiocyanate (FITC), SYTO9 green fluorescent nucleic acid stain (Thermo Fisher Scientific) or transformation with pEGFP (plasmid carrying the green fluorescent protein [GFP] gene; Table 1). After incubation at 37 °C for different times, wells were washed to eliminate unbound bacteria and PAMs with associated bacteria were detected by flow cytometry in a MACS Miltenyi cytometer (MILTENYI Biotec, Madrid, Spain). Assays were performed in duplicate and were repeated using PAM from different animals.

### Serum resistance assay

To test if VtaA2 was involved in serum resistance, an in vitro assay with rabbit serum was performed as described before [9] with some modifications. Briefly, overnight colonies of *E. coli* BL21 (pACYC184) and *E. coli* BL21 (pLGR-vtaA2) were used to prepare bacterial suspensions. Bacteria and serum were mixed in 96 well plates to give a final quantity of bacteria in the wells of 10^5^–10^6^ CFU and a final concentration of 50% serum. Mixtures were incubated at 37 °C for 30 min and 1 h. Tenfold serial dilutions of each well were made before and after incubation, and bacteria were quantified by plating onto LB-Cm30. The counts of viable bacteria before and after serum treatment were compared.

## Results

### VtaA2 is involved in attachment to animal proteins

**Figure 1:**
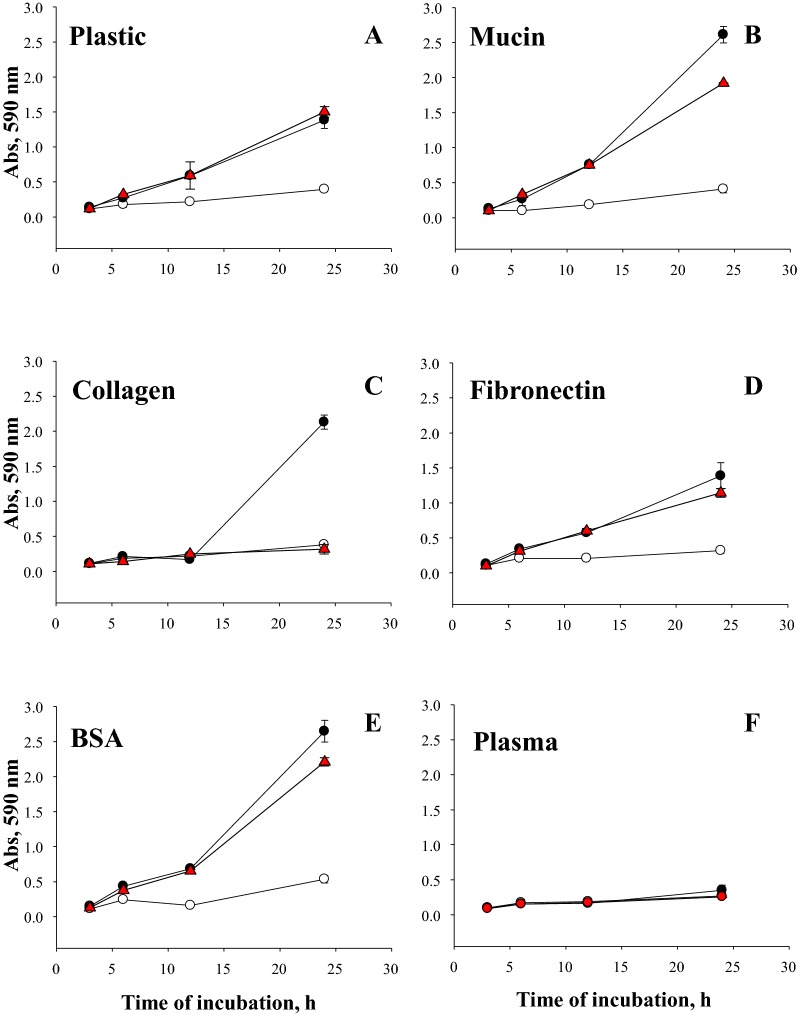
**Kinetics of attachment to different substrates of** ***E. coli*** **clones expressing VtaA2 and VtaA2OL**. Wells were coated with mucin (**B**), collagen (**C**), fibronectin (**D**), BSA (**E**), or plasma (**F**), or were left uncoated (plastic **A**). Plates were inoculated and incubated under static conditions. Attachment was measured at different time points. Black circles: BL21 (pLGR-vtaA2); red triangles: BL21 (pLGR-vtaA2OL); and white circles BL21 (pACY184) (control). Bars represent the standard deviation of the mean Abs_590_ from triplicate wells.

A fosmid containing *vtaA2* was used as template to amplify and subsequently clone *vtaA2* with its putative promotor region in pACYC184, yielding pLGR-vtaA2. *E. coli* BL21 (pLGR-vtaA2) showed a growth in LB-Cm30 similar to the *E. coli* with the empty plasmid pACYC184 (not shown). Compared to the empty plasmid control, BL21 (pLGR-vtaA2) exhibited more capacity to attach to plastic and to all the purified proteins tested (Figure 1), indicating that VtaA2 promoted attachment. Attachment to plastic (Figure 1A), mucin (Figure 1B), collagen (Figure 1C), fibronectin (Figure 1D) and BSA (Figure 1E) by the *vtaA2* clone was detected as soon as 6 h of incubation. However, no attachment to swine plasma was observed, indicating no ability of VtaA2 to bind to plasma components (Figure 1F).

### Collagen domains of VtaA2 are involved in collagen attachment

To examine the role of the collagen domains of VtaA2 in the attachment observed, a truncated version of *vtaA2* lacking the central portion of the gene that encoded the collagen domains was cloned in pACYC184 to yield pLGR-vtaA2OL. Similarly, BL21 (pLGR-vtaA2OL) displayed comparable level of attachment to plastic, mucin, fibronectin and BSA than the *E. coli* with the complete *vtaA2* (Figure 1). However, a reduction in the attachment to collagen was observed in the truncated VtaA2OL (Figure 1C), indicating that the collagen domains of VtaA2 may play a role in attachment to collagen through collagen-collagen interaction.

### VtaA2 does not promotes cell adhesion or invasion

**Figure 2:**
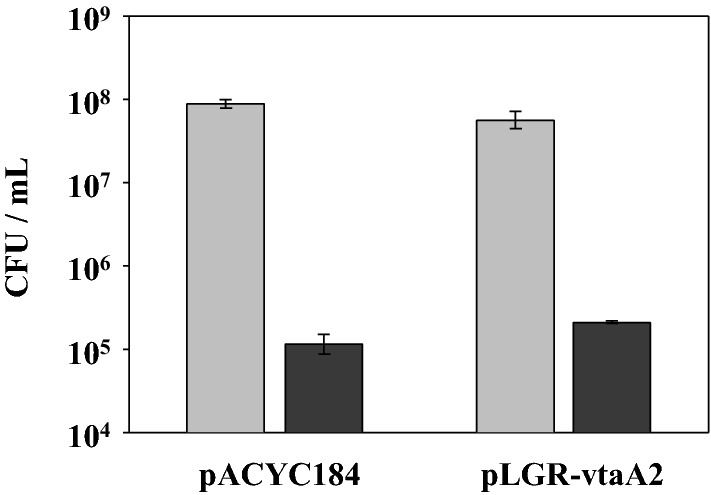
**VtaA2 has no role in the adhesion to lung epithelial cells A549 cells**. Adhesion of the clones *E. coli* BL21 (pLGR-vtaA2) and (pACYC184) to lung epithelial cells A549 after 1 h of incubation (black bars). Concentration of bacteria in the wells at the beginning of the assay are shown in gray bars. Bars represent the mean ± standard deviation of the concentration of bacteria from duplicate wells. Results are representative of 2 independent experiments.

To explore if VtaA2 could play a role in adhesion to epithelial cells, an assay with the pulmonar epithelial cell line A549 was performed. After 1 h of incubation to allow the attachment of the bacteria to the cells, no differences were observed between the control *E. coli* BL21 (pACYC184) and the clone carrying *vtaA2* (Figure 2), indicating that VtaA2 does not have a direct effect on bacterial adhesion to epithelial cells. In agreement with the lack of effect on cell adhesion, we did not detect a role of VtaA2 in cellular invasion (not shown). In fact, neither BL21 (pACYC184) nor BL21 pLGR-vtaA2 were able to invade epithelial A549 cells, and internalized bacteria were not detected in any of the cases.

### VtaA2 does not play a role in the interaction with porcine alveolar macrophages (PAMs)

**Figure 3:**
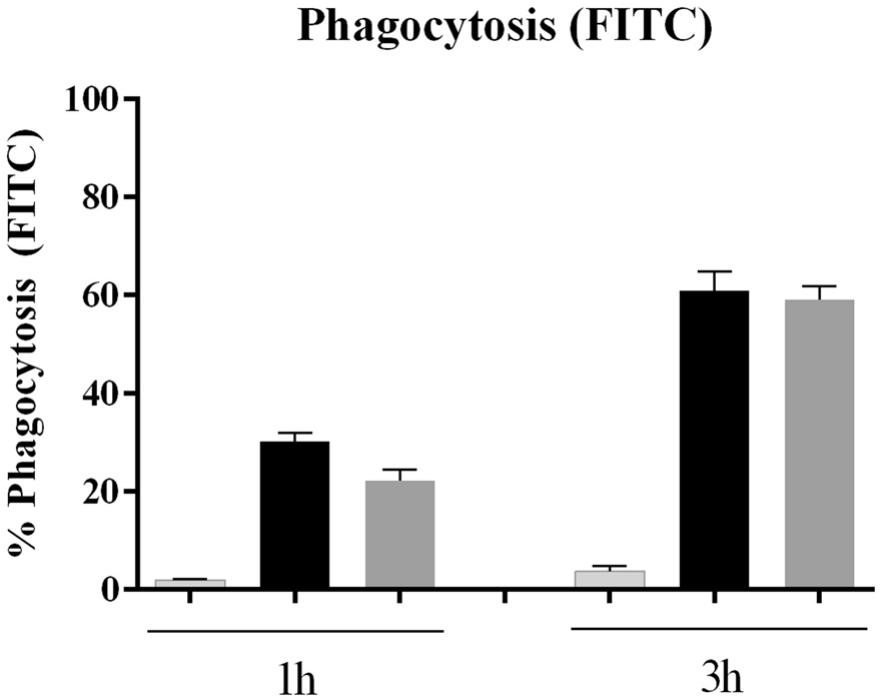
**VtaA2 has no role in the bacterial interaction with PAMs; bacteria stained with FITC.** *E. coli* BL21 clones (pLGR-vtaA2; dark grey bars) and empty vector pACYC184 (black bars) were stained with FITC, incubated for 1 h or 3 h with PAMs and the interaction with the macrophages was analyzed by flow cytometry. Results are shown as the percentage of fluorescent macrophages. Macrophages without bacteria were included as negative (light grey bars). Bars represent the mean ± standard deviation.

**Figure 4:**
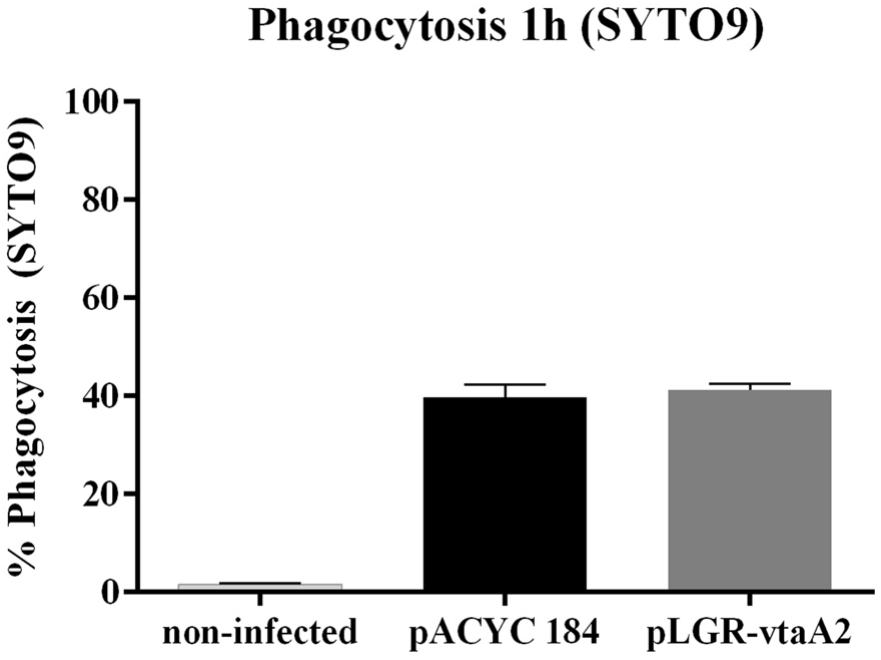
**VtaA2 has no role in the bacterial interaction with PAMs; bacteria stained with SYTO9.** *E. coli* BL21 clones (pLGR-vtaA2; dark grey bars) and empty vector pACYC184 (black bars) were stained with SYTO9, incubated for 1 h with PAMs and the interaction with the macrophages was analyzed by flow cytometry. Results are shown as the percentage of fluorescent macrophages. Macrophages without bacteria were included as negative (light grey bars). Bars represent the mean ± standard deviation.

Assays to evaluate the role of VtaA2 in phagocytosis resistance were performed. Unfortunately, fluorescent labelling of *E. coli* BL21 (pLGR-vtaA2) and the control *E. coli* was challenging due to differences in staining between them. On one hand, BL21 (pLGR-vtaA2) transformed with pEGFP did not consistently express GFP, unlike the empty plasmid control, making this labelling inadequate for this study. On the other hand, FITC did not label properly the control BL21 (pACYC184), and 5 times more compound was needed to reach similar fluorescence intensity than the BL21 expressing VtaA2. When FITC-labelled bacteria were used, similar association with PAMs was observed in the *vtaA2* clone and the control (Figure 3). To confirm these results, the nucleic acid stain SYTO9 was used to label the bacteria. Equivalent mean of fluorescence intensity in the bacteria was obtained with this dye (BL21 control: 12.9; BL21 with *vtaA2*: 10.63). Again, the percentage of PAMs associated with the fluorescent *vtaA2* clone was similar to the percentage of PAMs with the control bacteria (Figure 4), indicating that VtaA2 does not play a role in phagocytosis.

Additionally, attachment to PAMs was examined by incubation of the clones with PAMs on ice during 1 h. High levels of attachment to the surface of PAMs were found and no differences were detected in *E. coli* BL21 (pLGR-vtaA2) and the empty plasmid control (not shown), indicating that VtaA2 does not to play a role in adhesion to macrophages.

### VtaA2 is not involved in serum resistance

Both BL21 (pACYC184) and BL21 (pLGR-vtaA2) showed similar survival rate after incubation with rabbit serum. A reduction of 5 log was observed for both clones after 30 min of incubation with 50% serum, and no recovery of live bacteria was detected after 1 h of incubation (not shown). Therefore, this suggests that VtaA2 has no effect on evasion of killing by serum complement.

### Expression of VtaA2 and VtaA2OL

**Figure 5:**
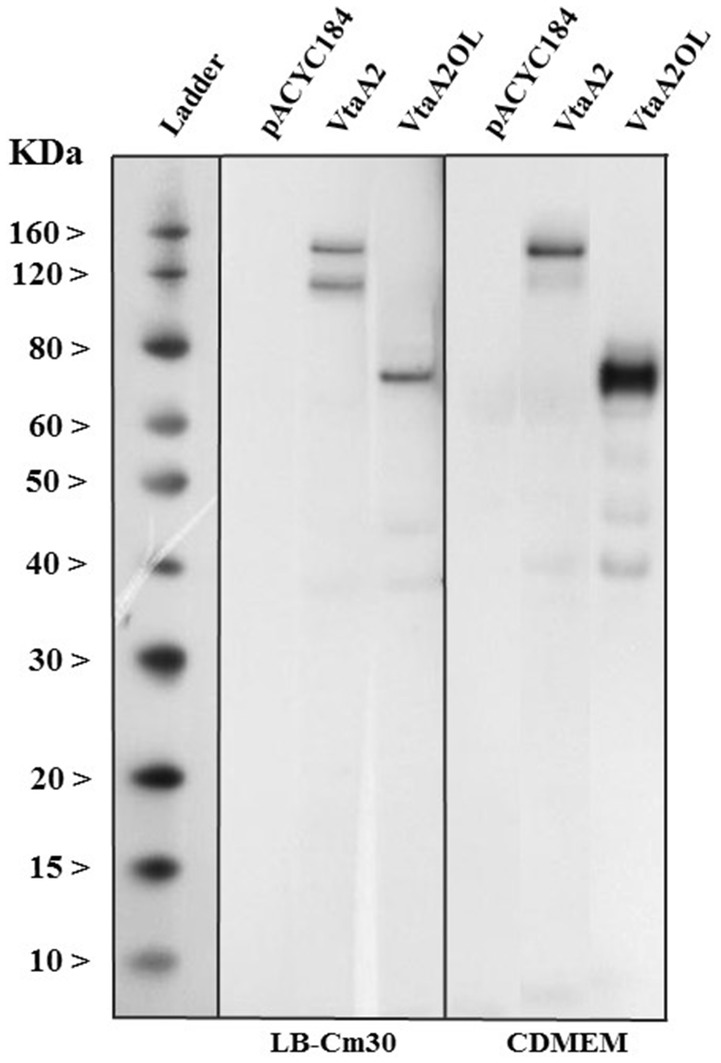
**VtaA2 and VtA2OL are produced under in vitro conditions.** Detection by immunoblot of VtaA2 and its truncated version VtaA2OL from *E. coli* BL21 (pLGR-vtaA2) and *E. coli* BL21 (pLGR-vtaA2OL) respectively. Both proteins were extracted from bacterial pellets grown in LB-Cm30 or incubated with CDMEM. Molecular size standards in kDa are shown on the left. Protein extraction from *E. coli* BL21 transformed with pACYC184 empty plasmid was used as negative control of expression.

Proteins of approximately the expected sizes of VtaA2 (120.99 kDa) and VtaA2OL (75.61 kDa) were detected in the bacterial pellet of the corresponding clones grown under the conditions used in the functional assays (Figure 5), confirming that the lack of phenotype found in cell adhesion, invasion or phagocytosis were not due to the lack of protein production.

## Discussion

Bacterial pathogens adhere to epithelial cells or host proteins in order to prevent clearance by the host. Adhesion is achieved by adhesins, which are bacterial surface structures that confer ability to bind to surrounding cells, proteins or tissues. Virulent strains of *H. parasuis* have the ability to overcome the host clearing system, and after an initial colonization of the upper respiratory tract, can reach the lower respiratory tract, multiply and spread systemically [27]. However, how *H. parasuis* infection is established at systemic sites is not fully understood yet. Some VtaAs, including VtaA2, are associated with virulent strains of *H. parasuis* [17]. The involvement of VtaA2 in *H. parasuis* virulence was supported by its up-regulation during lung infection [24], but until now no specific function was assigned to this protein. Here we have shown that VtaA2 is able to promote attachment to several host molecules in a time-dependent manner. The ability of VtaA2 to bind to mucin could be an indication of the role of this protein in the initial attachment to the upper respiratory tract. Later, in the lung, VtaA2 could still promote attachment, while VtaA8 and VtaA9, which are also up-regulated during lung infection [24], would participate in phagocytosis resistance [18]. In addition, the capacity of VtaA2 to bind to ECM molecules suggest that VtaA2 is also important once *H. parasuis* spreads to internal organs. Therefore, we could hypothesize a dual role of VtaA2 in *H. parasuis* pathogenesis, in the initial colonization of the respiratory tract and in the posterior invasive infection.

Mucin is the major structural component of mucosa secretion that overlays the upper respiratory tract of swine. Mucins are a family of heavily O-glycosylated proteins that are the major organic components of the mucus layer, the protective layer covering the epithelial cells in many human and animal organs [28]. Pathogens that infect mucosal surfaces share two main goals: (1) to overcome the mucus barrier and (2) to interact with the underlying epithelial cells and cause disease [29]. Mucus layers consist mainly of gel-forming mucin-type glycoproteins [28]. Most mucins have a high sialic acid content. Interestingly, systemic isolates of *H. parasuis* present the sialyltransferase-encoding gene *lsgB* gene, which is involved in the terminal sialylation of the lipooligosacharide (LOS), with roles in host cell adhesion and serum resistance [30, 31]. LOS sialylation is a molecular mimicry mechanism extensively used by bacteria to evade the host immune system. We could hypothesize that, by binding to mucin through VtaA2, *H. parasuis* could enhance its interaction to mucin, facilitating LOS sialyation by using mucin as source of sialic acid, and promoting *H. parasuis* virulence. In addition, prior infection of piglets by *Bordetella bronchiseptica*, which induces an accumulation of mucus [32], results in an increased colonization by *H. parasuis* [33]. VtaA2 could play a key role in such scenario by promoting attachment to mucin in the abundant mucus.

Inflammatory responses due to multiple pathogens can disrupt the mucus layer and cause epithelial damage in the airway mucosa, allowing access to intruding bacteria to the underlying epithelium and to ECM proteins, and explaining the exacerbation of clinical signs [34, 35]. Virulent strains of *H. parasuis* can be detected in pig lung, nasal turbinates, trachea including the submucosa [36], indicating that, as other respiratory pathogens, *H. parasuis* may have the capacity to bind and degrade ECM proteins in order to adhere to and invade host tissues [37]. Collagens are the most abundant proteins in the lung ECM [38]. Collagen can be associate with cartilage, including trachea, present in the connective tissues or as part of the basal lamina that anchors epithelial cells to the mesenchyme [39]. The attachment of the host cells to components of the ECM, such as collagen, is also mediated by fibronectin [40]. Multiple bacterial fibronectin and collagen binding proteins have been described, including trimeric autotransporters [41–43]. Therefore, expression of the trimeric autotransporter VtaA2 in the bacterial surface could be a key factor for Glässer’s disease progress by attachment to both collagen and fibronectin.

Interestingly, VtaA2OL showed a reduced capacity to bind to collagen, suggesting that the loss of these collagen domains in VtaA2 are essential for collagen attachment, by collagen-collagen interaction. On the other hand, VtaA2 includes other binding domains to host molecules since the attachment to other proteins, such as mucin or fibronectin, is not affected when the collagens domains were removed. In fact, other domains with adhesion function previously described in VtaAs [17] are characteristic of cell surface adhesion molecules from other pathogenic bacteria [44, 45]. Further characterization of the VtaA2 structure would be needed to elucidate other functional domains, conformational structures or other factors that can affect the binding affinity for host receptors.

With the dramatic rise of antimicrobial resistances and the corresponding threat to human and animal health, it is crucial to find alternative approaches to block the ability of bacteria, such as *H. parasuis*, to cause disease at the early stages of infection with the design, for instance, of anti-adhesion vaccines.

## Abbreviations

CDMEM: complete DMEM
Cm: chloramphenicol
DMEM: Dulbecco’s modified Eagle’s medium
ECM: extracellular matrix
FITC: fluorescein isothiocyanate
GFP: green fluorescent protein
LB: Luria–Bertani
LOS: lipooligosaccharide
PAMs: porcine alveolar macrophages
PBS: phosphate buffered saline
VtaA: virulence-associated trimeric autotransporter
